# Histone methyltransferase WHSC1 inhibits colorectal cancer cell apoptosis via targeting anti-apoptotic BCL2

**DOI:** 10.1038/s41420-021-00402-6

**Published:** 2021-01-19

**Authors:** Yu Wang, Liming Zhu, Mei Guo, Gang Sun, Kun Zhou, Wenjing Pang, Dachun Cao, Xin Tang, Xiangjun Meng

**Affiliations:** 1 Departments of Gastroenterology, Shanghai Ninth People’s Hospital, Shanghai Jiaotong University School of Medicine, Shanghai, China; 2 Departments of Geriatrics, Shanghai Ninth People’s Hospital, Shanghai Jiaotong University School of Medicine, Shanghai, China

**Keywords:** Colon cancer, Apoptosis, Epigenetics

## Abstract

WHSC1 is a histone methyltransferase that facilitates histone H3 lysine 36 dimethylation (H3K36me2), which is a permissive mark associated with active transcription. In this study, we revealed how WHSC1 regulates tumorigenesis and chemosensitivity of colorectal cancer (CRC). Our data showed that WHSC1 as well as H3K36me2 were highly expressed in clinical CRC samples, and high WHSC1 expression is associated with poorer prognosis in CRC patients. WHSC1 reduction promoted colon cancer cell apoptosis both in vivo and in vitro. We found that B cell lymphoma-2 (BCL2) expression, an anti-apoptotic protein, is markedly decreased in after WHSC1 depletion. Mechanistic characterization indicated that WHSC1 directly binds to the promoter region of *BCL2* gene and regulate its H3K36 dimethylation level. What’s more, our study indicated that WHSC1 depletion promotes chemosensitivity in CRC cells. Together, our results suggested that WHSC1 and H3K36me2 modification might be optimal therapeutic targets to disrupt CRC progression and WHSC1-targeted therapy might potentially overcome the resistance of chemotherapeutic agents.

## Introduction

Colorectal cancer (CRC) is 3rd most commonly diagnosed malignancy and the 2nd leading cause of cancer death globally^1,2^. Until now, CRC remains a major cause of cancer mortality worldwide, bringing about serious threats to human health^3^. Within colorectal cancer cells, BCL2 is well known to act to suppress the induction of apoptosis^4^. In the colon cancer patient tissue BCL2 is often abnormally highly expressed, and higher frequencies of BCL2-positive cells are linked with lower overall rates of apoptosis^5^. It is therefore vital that we understand how cancer cells modulate BCL2 expression to subvert apoptosis, in order to develop new treatments for this deadly disease.

Traditionally, epigenetic regulation refers to diverse and reversible chemical modifications on DNA or histones. Among these diverse modifications, histone methylation at different sites lead to increased or inhibited gene expression, which regulate gene expression in a way independent to genome changes^6,7^. Epigenetic regulation is widely reported to participate in the development and progression of CRC^8^, and histone methyltransferases are key players in this regulation. CRC evolves as a result of the stepwise accumulation of a series of epigenetic alterations, in recent studies, the pathophysiological contribution of epigenetic regulation in CRC has attracted considerable attention^9,10^. Take for instance, targeting histone methyltransferase EZH2 (zeste homolog 2) significantly alleviates intestinal inflammation, EZH2 is correlated with colorectal cancer stage and prognosis, linked to CRC tumorigenesis^11–13^.

The histone methyltransferase WHSC1 facilitates dimethylation of lysine 36 on the H3 histone (H3K36me2), which activates transcription at modified loci^14^. In the published results, WHSC1 knockout mice showed cardiac developmental defects^15^, and WHSC1 is necessary for B-cell development^16^. In addition, WHSC1 also has been shown to play important roles in cancer, it is highly expressed in many human cancers and affects the cell cycle by regulating the WNT signaling pathway^17^. WHSC1 also directly interacts with NF-κB to activate additional target genes that have been linked with improved survival of prostate cancer cells^18^. WHSC1 promotes tumorigenesis in salivary adenoid cystic carcinoma by regulating expression of stemness-related genes MYC^19^. Moreover, in prostate cancer, WHSC1 control cancer metastasis by targeting transcription factor TWIST1 and mTORC2 signaling^20,21^.

Here in our study, we firstly reported that WHSC1 and H3K36me2 levels were elevated in human CRC, and WHSC1 inhibited colon cancer cell apoptosis by directly binding to the promoter region of BCL2. WHSC1 depletion inhibited BCL2 expression and suppressed CRC tumorigenesis both in vitro and in vivo. WHSC1 reduction promotes the Oxaliplatin sensitivity in CRC cells. Our findings thus demonstrate that WHSC1 activity may be a potential therapeutic target in colon cancer.

## Results

### WHSC1 expression is upregulated in human CRC

**Fig. 1:**
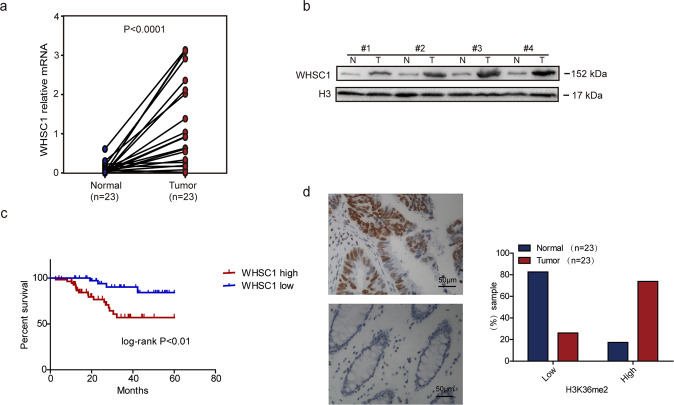
Elevated WHSC1 expression in human CRC. **a** WHSC1 mRNA levels in 23 matched tumor and paracarcinoma tissues (paired *t-*test, *P* < 0.001). **b** Immunoblotting analysis of WHSC1 expression in four matched CRC tissues (T) and adjacent noncancerous tissues (N). **c** Kaplan–Meier plot of overall survival of CRC patients based on WHSC1 levels. A log-rank test was used for statistical analysis. **d** Expression of H3K36me2 was assessed by immunohistochemistry. H3K36me2 staining indexes using a 10-point quantification are shown.

In order to determine the role of WHSC1 in colorectal cancer, we initially investigated WHSC1 expression in CRC patient by quantitative reverse-transcriptase PCR (RT-qPCR). WHSC1 expression was elevated significantly in cancerous tissues relative to normal counterparts (Fig. 1a). Next, Western blots showed that WHSC1 protein levels were both increased in these CRC tumors relative to normal controls (Fig. 1b). Simultaneously, we used immunohistochemical detection of a colorectal cancer tissue microarray to analyze WHSC1 expression in 97 archived metastasis-free human CRC specimens by immunohistochemistry (IHC). Our results demonstrated that patients with high WHSC1 expression level showed shorter metastasis-free survival (MFS) times than patients with low levels of WHSC1, which indicated that WHSC1 might be associated with the risk of CRC progression and could be an independent predictor of MFS (Fig. 1c). In addition, H3K36me2 levels were significantly elevated in CRC tumor tissue compared with normal counterparts as assessed by immunohistochemistry (Fig. 1d). Together, these data suggested WHSC1 as a potential CRC biomarker, and a possible causal role of WHSC1 in the development of CRC.

### WHSC1 reduction inhibits tumorigenesis in CRC lines

**Fig. 2:**
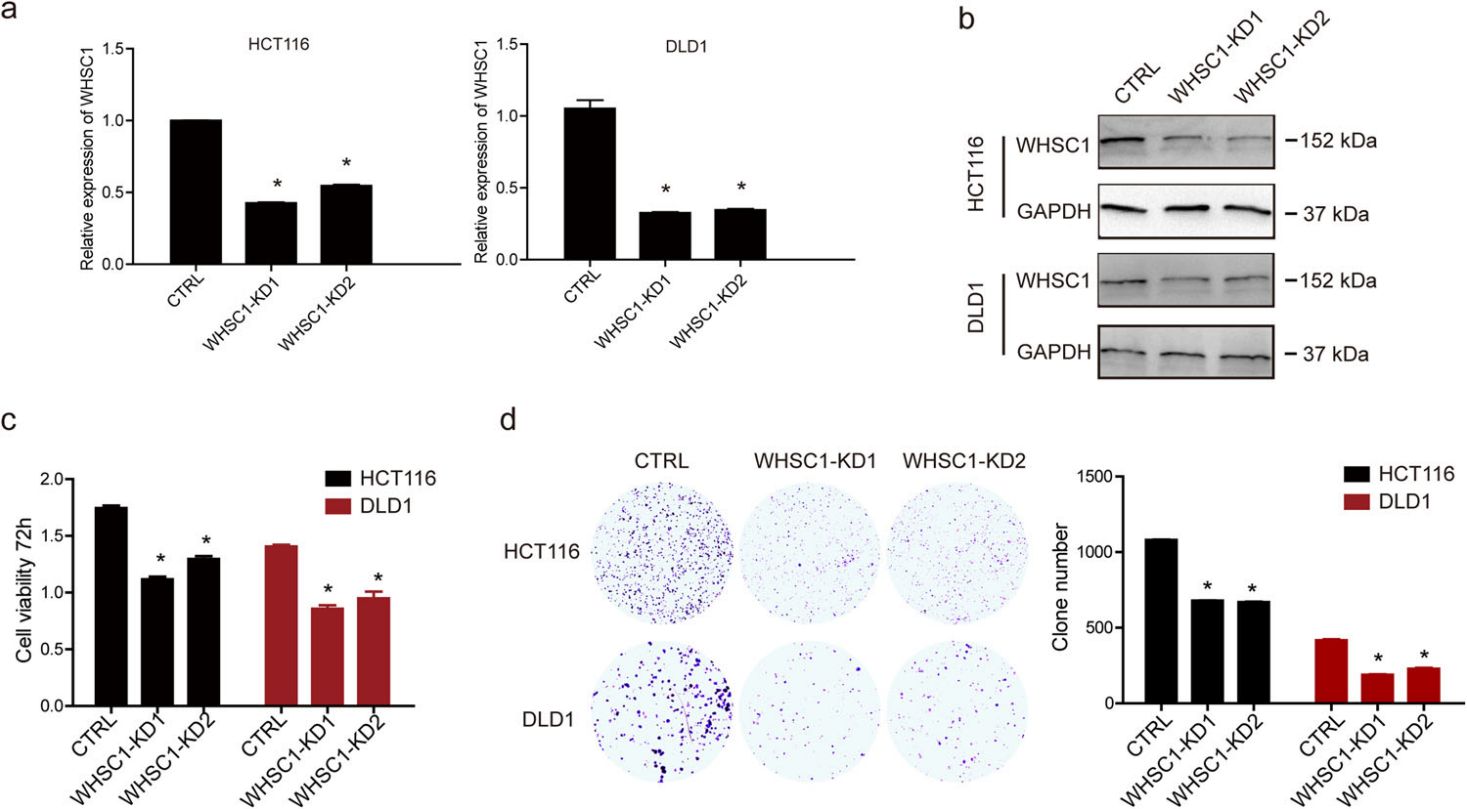
Knock down WHSC1 inhibits tumorigenesis in CRC cells. **a** qPCR analysis of the indicated mRNA in control and WHSC1 knockdown cell lines. **b** Immunoblotting analysis of WHSC1 in DLD-1 and HCT116 cells transduced with two specific shRNA. **c**, **d** CCK-8 assays (**c**) and colony formation assays (**d**) in parental and WHSC1 knock down cells. The quantitation results are shown in the right panels.

To assess WHSC1 function in CRC, we knocked down WHSC1 in two CRC cell lines, HCT116 and DLD1, using pLVX-shRNA1 plasmid with control shRNA and two independent WHSC1 shRNA constructs. Both mRNA and protein levels of WHSC1 were confirmed to be significantly decreased after shRNA transduction in both two cell lines (Fig. 2a, b). Reduced WHSC1 significantly inhibited tumor cell growth ability as assessed by CCK-8 and colony formation assay (Fig. 2c, d). These data suggested that WHSC1 benefited CRC progression in vitro.

#### WHSC1 ablation increases apoptosis of CRC cells

**Fig. 3:**
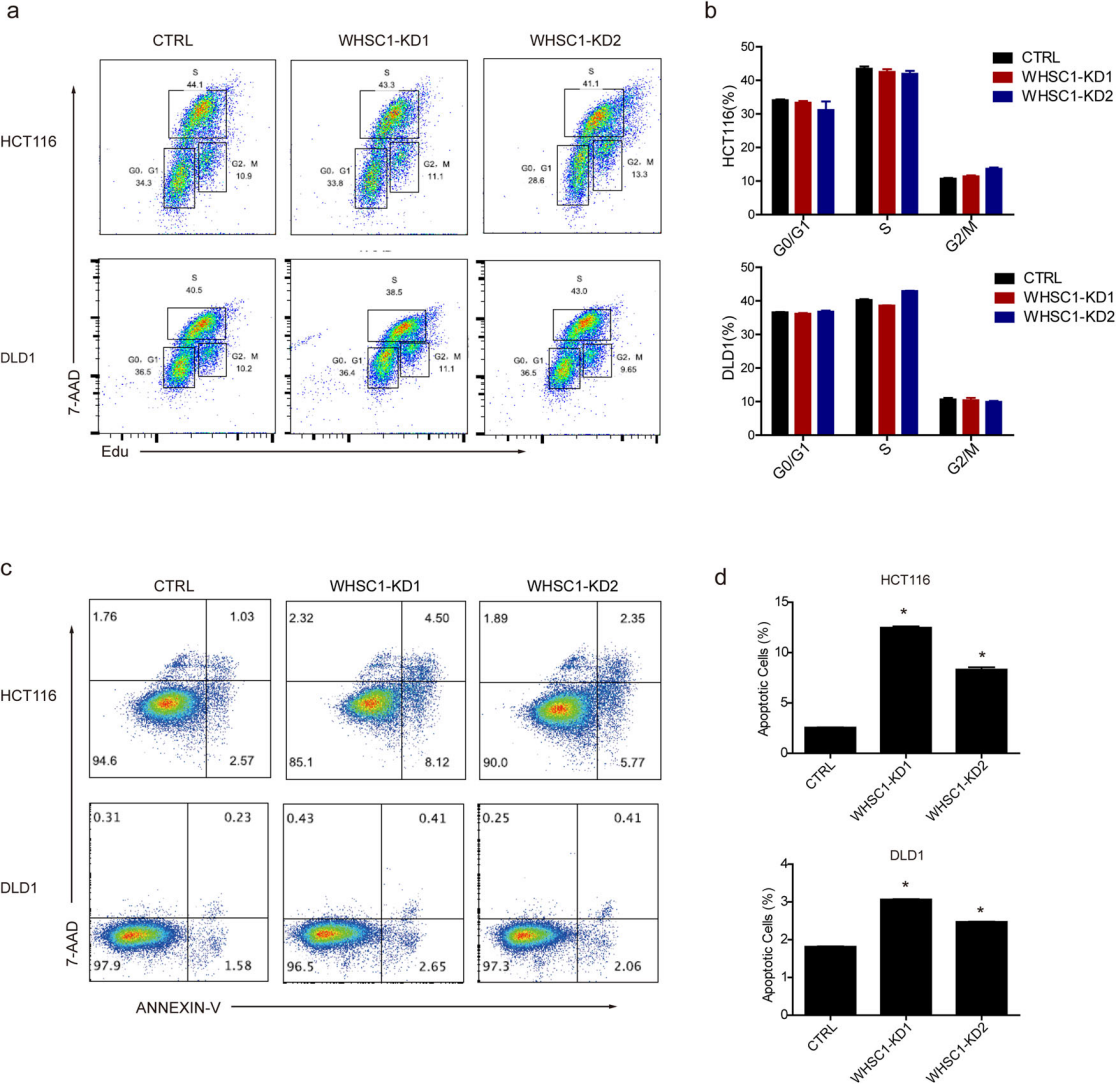
WHSC1 ablation promotes CRC cell apoptosis. **a** Profiling of the cell cycle distribution of HCT116 and DLD1 cells transiently expressing shCon, shWHSC1-1, or shWHSC1-2. **b** Quantitative analyses of the cell cycle distribution as described in (**a**), assessed by two-sided Student’s *t* test. **c**, **d** Flow cytometry profiling and quantification of Annexin-V/7-AAD-stained cells transiently expressing shCon, shWHSC1-1, or shWHSC-2. All data represent the mean ± S.D. of three independent experiments, *P < 0.05, **P < 0.01.

Apoptosis and cell cycle are two important factors that relative to CRC progression. We therefore assessed cell apoptosis by Annexin V-7AAD assay and cell cycle progression by Edu assay in WHSC1-knockdown (WHSC1-KD) colon cancer cells. Data revealed that cell cycle progression seemed to be intact after knocking down WHSC1 (Fig. 3a, b). But WHSC1 knockdown significantly increased apoptosis in HCT116 and DLD1 cell (Fig. 3c, d). The above data indicated that the inhibited cell viability in WHSC1-deficient colon cancer cells were due to increased cell apoptosis rather than decreased cell proliferation.

### WHSC1 depletion inhibits CRC development in vivo by promoting apoptosis

**Fig. 4:**
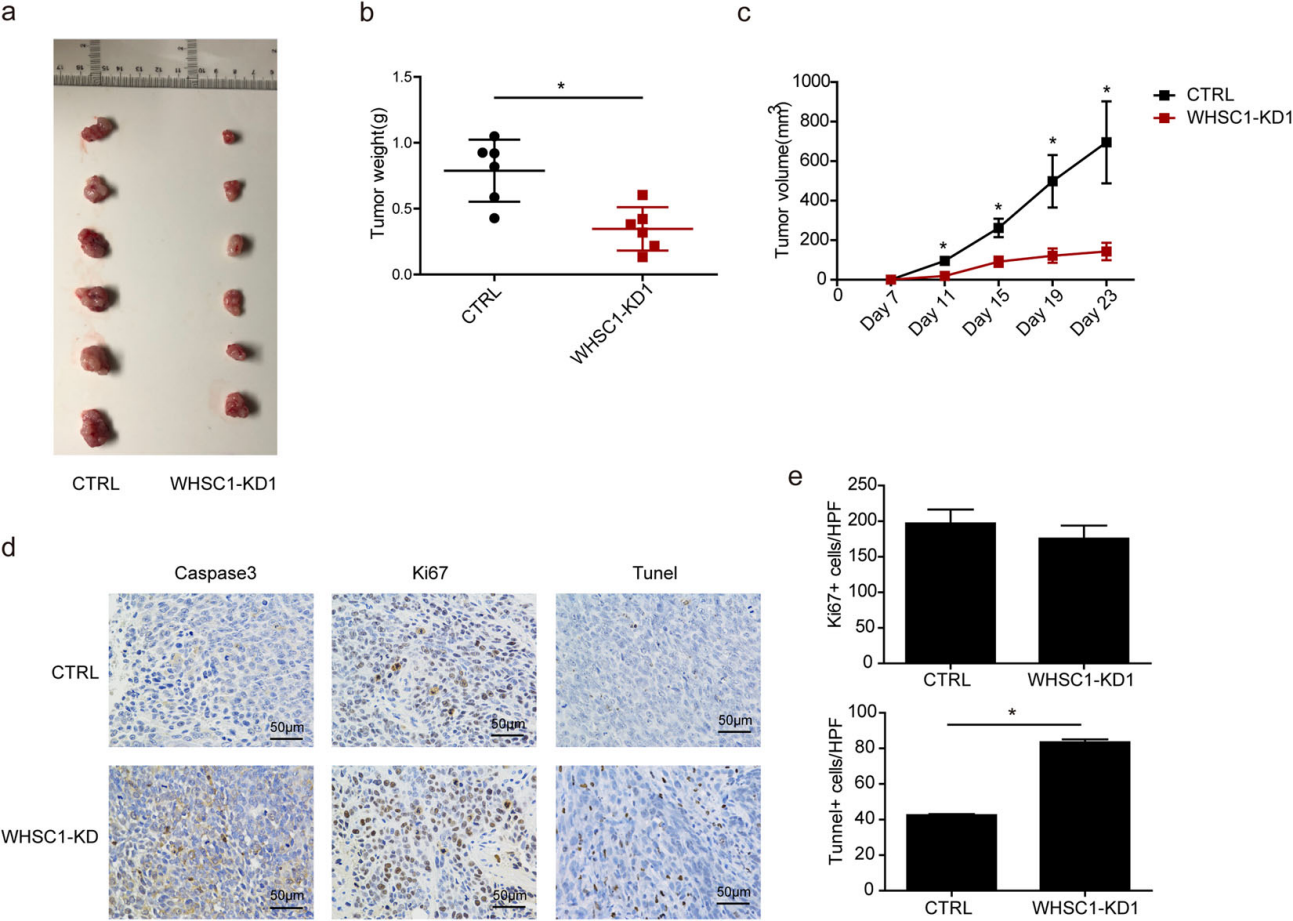
WHSC1 reduction suppresses CRC cell growth and promotes apoptosis in vivo. **a** Tumor growth in mice injected with HCT116 cells stably expressing shWHSC1-1 or the control. **b**, **c** Tumor volume (**b**) and volume weight (**c**) in mice injected with HCT116 cells stably expressing control or shWHSC1-1 (*n* = 6). **d**, **e** Immunohistochemistry results for TUNEL and caspase3 assays of tumors derived from HCT116 cells stably expressing control or shWHSC1-1.

We next used mouse xenograft models to investigate the importance of WHSC1 in CRC development in vivo. HCT116 cells were transfected with WHSC1 or control shRNA and stable cell lines were established by puromycin screening. Cells were then subcutaneously implanted into left armpit of nude mice (6 mice per group). Tumor volumes were monitored, and mice were euthanized at 3 weeks post implant to measure tumor weight. We found that WHSC1 knock-down lead to delayed tumor progression and decreased tumor weight as compared with control CRC cells (Fig. 4a–c). Immunohistochemistry assay revealed that there were more TUNEL-positive and caspase-positive cells in WHSC1-knockdown tumor group compared with control group (Fig. 4d, e). However, there were no significant difference between two groups in ki67-positive cells (Fig. 4d, e). These results indicated that WHSC1 promotes tumor development by inhibiting apoptosis in vivo.

### WHSC1 promotes anti-apoptotic BCL2 expression

**Fig. 5:**
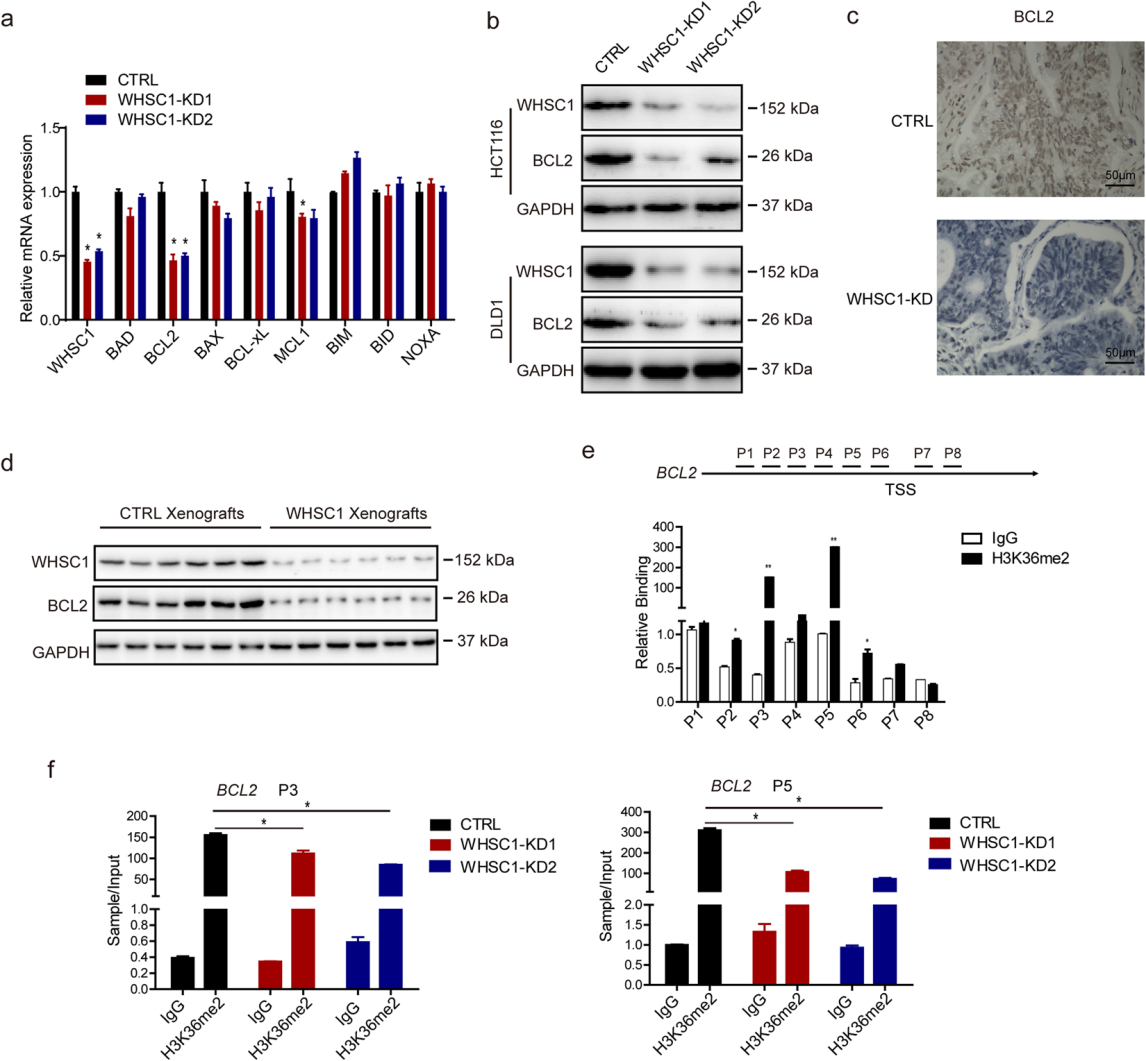
WHSC1 promotes BCL2 transcription through upregulating H3K36me2 modification on the gene loci. **a**, **b** The mRNA expression of BCL2 family genes in the WHSC1-knockdown HCT116 cells compared with control cells. **b** The BCL2 protein level in the WHSC1 knock down Hct116 and DLD1 cells compared with control cells. **c**, **d** Immunohistochemical and Western detection of BCL2 expression WHSC1 knock down xenografted tumor tissue compared with control tissue. **e** ChIP-qPCR analysis of the binding of H3K36me2 to the BCL2 promoter in HCT116 cells. **f** ChIP-qPCR analysis for anti-H3K36me2 along with control IgG at the BCL2 promoter loci in control or WHSC1 knock down HCT116 cells.

In order to explore the mechanism how WHSC1 inhibits apoptosis in CRC, first we checked mRNA expression of BCL2 family genes in control and WHSC1-KD CRC cells. We found that the BCL2 expression were markedly decreased after WHSC1 depletion (Fig. 5a). However, other BCL2 family genes associated with apoptosis, such as BAD, BAX, BIM, BID, BCL-XL, and NOXA, were intact in WHSC1-KD cells, only a slight decrease in MCL1 expression was observed (Fig. 5a). Protein level of BCL2 was also significantly reduced in WHSC1-KD HCT116 and DLD1 cell lines (Fig. 5b). Consistently, there were also significantly decreased BCL2 expression level in WHSC1-knockdown xenografted tumor tissue compared with control xenografted tumor as assessed by immunohistochemistry and Western blot (Fig. 5c, d). These findings suggest that WHSC1 might mediate cell apoptosis of CRC by controlling BCL2 expression.

### WHSC1 mediates H3K36me2 modification at transcriptional initiation region of BCL2

Since WHSC1 is well known for its histone methyltransferase activity, and H3K36me2 is associated with active gene transcription^14^. We hypothesized that if WHSC1 bind to transcriptional initiation region of *BCL2* gene and directly induced H3K36 dimethylation so as to promote *BCL2* transcription. Chromatin immunoprecipitation (ChIP) was performed in CRC cells to assess this possibility. WHSC1 was significantly enriched at 1 to 1.5 (P3) kilobases and 2 to 2.5 kilobases (P5) upstream of the transcription start site (TSS) (Fig. 5e). And levels of H3K36me2 modification at the loci of P3 and P5 in WHSC1-knockdown CRC cells were significantly lower than that in control cells (Fig. 5e). Therefore, our results indicated that *BCL2* is a direct target gene of WHSC1, WHSC1 promoting *BCL2* transcription by controlling the H3K36 dimerization modification in its transcription initiation region, so as to regulate colon cancer cell apoptosis.

### WHSC1 reduction increases chemosensitivity of oxaliplatin and 5-fluorouracil in CRC cells

**Fig. 6:**
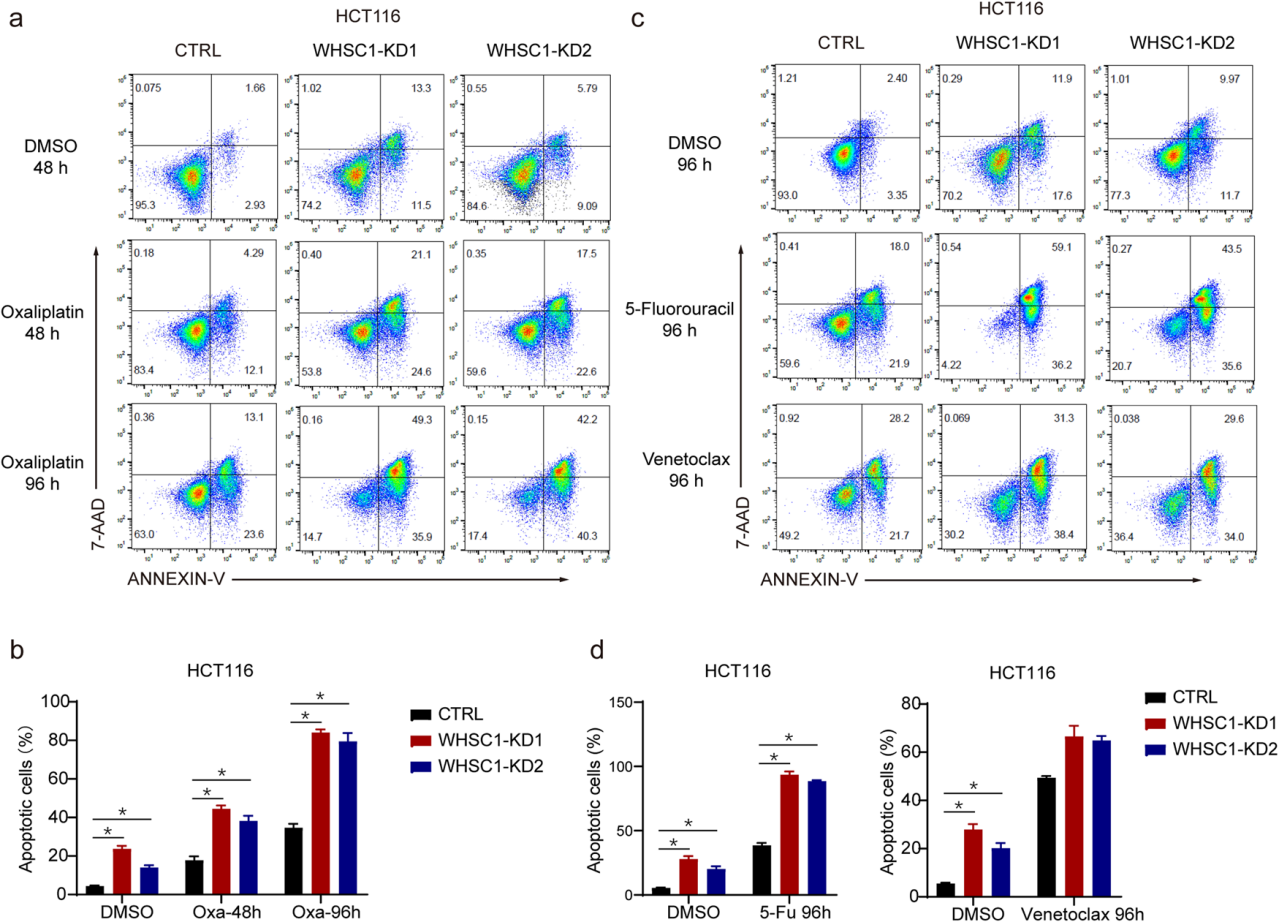
WHSC1 reduction increases chemosensitivity of oxaliplatin and 5-fluorouracil in colon cancer cells. **a** Control and WHSC1-KD CRC cells were treated with oxaliplatin (Oxa, 20 μM) for indicated time course. The percentage of cells entering apoptosis was determined by flow cytometry using FITC-labeled Annexin V staining. **b** Control and WHSC1-KD CRC cells were treated with 5-fluorouracil (5-Fu, 20 μM) or venetoclax (10 μM), and Annexin V-7AAD staining was performed to determine apoptotic cells. **c**, **d** Quantitative results for **a** and **b**.

In addition to cancer cell apoptosis, BCL2 was also reported to confer chemoresistance in colorectal cancer^5^. What’s more, BCL2 family genes were used to establish a model to predict responses to chemotherapy in colorectal cancer^22^. Oxaliplatin and 5-fluorouracil are commonly used as standard first-line chemotherapeutic drugs for colon cancer^23^. We further investigate if WHSC1 and H3K36me modification play a role in oxaliplatin resistance. WHSC1-KD and control CRC cells were treated with 20 μM oxaliplatin for indicated time course. Results revealed that oxaliplatin-induced apoptosis was markedly higher in WHSC1-KD cells compared with that in control cells, and long-term treatment of drugs made the synergistic effect more significant (Fig. 6a, b). Similarly, after treatment with the same concentration of 5-fluorouracil, more CRC cells undergo apoptosis in WHSC-KD cells compared with those in control cells (Fig. 6c, d). Venetoclax is a selective BCL2 inhibitor, which is approved in numerous countries for front-line treatment of acute myeloid leukemia (AML) and chronic lymphocytic leukemia (CLL)^24,25^. We compared the response to venetoclax in control and WHSC1-KD CRC cells. Interestingly, venetoclax treatment in combination with WHSC1 reduction results in scant increase in percentage of apoptotic cells compared with control CRC cells treated with venetoclax. We conjectured that the downregulated BCL2 expression in WHSC1-KD CRC cells probably lead to the lack of synergistic effect on BCL2-specific inhibitors, or to some extent, it was due to the fact that WHSC1 and venetoclax targeted the same molecule. Overall, we summarized that inhibition of WHSC1 expression significantly increased the chemosensitivity of first-line chemotherapeutic drugs oxaliplatin and 5-fluorouracil in CRC cells.

## Discussion

BCL2 is highly expressed in many colon cancer patients to inhibit apoptosis^4,26^. Therefore, the specific targeting of BCL2 to promote apoptosis may be a viable therapeutic approach. Here we show that the methyltransferase WHSC1 is also highly expressed in colon cancer samples, and WHSC1 bind to *BCL2* gene promoter, changing the H3K36me2 modification state to increase its transcription. WHSC1 depletion reduces BCL2 expression, leading to elevated cell apoptosis during CRC progression.

So far, WHSC1 importance in colon cancer progression is rarely understood. BCL2 is one of the most known anti-apoptotic factors and found to be highly expressed in a variety of solid tumors relating to tumorigenesis^27–29^. So our results suggest a close relationship between the epigenetic regulator, WHSC1, and key intracellular oncogenes. WHSC1 knockdown significantly decreased the H3K36me2 modification level in *BCL2* gene locus, leading to inhibition of transcription of this key oncogene. We believe that the regulation of *BCL2* gene expression was an important but may not all effect of WHSC1 in tumorigenesis of CRC. WHSC1 has previously been linked to transcription initiation^14^, but whether WHSC1 also regulates other important genes associated with colon cancer necessitates ChIP-seq to verify.

Epigenetic alterations have a major role in a subset of CRCs. Although we have established the oncogenic role of WHSC1 in CRC cell apoptosis, we did not determine which oncogenic insults led to elevated WHSC1 expression in CRC cells. In a previous study, it was reported that increased AKT activity directly phosphorylates WHSC1 at S172 to promote its stability, preventing WHSC1 degradation, and lead to prostate cancer metastasis^21^. Previous evidence has indicated that the PI3K/Akt signal pathway activates signals for cell survival and cell proliferation in CRC^30^. AKT activation was inhibited by lipid phosphatase activity of a major tumor suppressor PTEN (phosphate and tension homology deleted on chromsome ten) and played an important role in carcinogenesis of CRC^31^. In our study, it was found that PTEN expression was significantly downregulated in colon cancer tissues (data not shown), so we suspected that this may be a cause of elevated WHSC1 expression in colon cancer cells.

In addition to transcriptional regulation, histone methyltransferases may also modify non-histone molecules. Histone methyltransferase enhancer of zeste homolog 2 (EZH2) was found to be able to catalyze K49 dimethylation of STAT3^32^, which is crucial for the expression of many IL-6–dependent genes. SET domain–containing protein 6 (SETD6) was identified as a protein lysine methyltransferases (PKMT) that monomethylated RelA at Lys310, which rendered RelA inert and attenuated RelA-driven transcriptional programs, including inflammatory responses in primary immune cells^33^. Whether WHSC1 can act similarly on non-histone proteins remains to be established.

In conclusion, we have demonstrated that WHSC1 is highly expressed in colon cancer and that it regulates BCL2 expression and cell apoptosis of CRC. This study elucidates a new epigenetic regulation mechanism during CRC progression. So it suggested that inhibiting WHSC1 enzymatic activity and H3K36 methylation state to promote colon cancer cell apoptosis, might be a new approach to therapeutic CRC treatment to be achieved.

## Materials and methods

### Cell lines, plasmids, reagents, and cell transduction

The colon cancer cell lines HCT116 and DLD-1 were bought from the American Type Culture Collection. DMEM supplemented with 10% fetal bovine serum (Gibco) was used to grown all cells. The FuGENE^®^ HD Transfection Reagent (Promega) was used for all transfections in accordance with manufacturer’s instructions. For WHSC1 knockdown, a WHSC1-specific shRNA was cloned into the pLVX-shRNA1 plasmid (Clontech). WHSC1-shRNA1: GTGCCAATAACACGTCCACT, WHSC1-shRNA2: GCCCTTCGCAGTGTTTGTCT. For a negative control, a scrambled sequence was used. Packaging was conducted with a three plasmid-system with psPAX2 and pMD2G. Lentiviral supernatant was used to infect cell lines, which were then selected for two weeks with 2 mg/mL puromycin.

### Human specimen analysis

Twenty-three colon cancer and paracancerous specimens were taken from CRC patients admitted to Shanghai Ninth People’s Hospital between 2010 and 2014. Written informed consent was obtained from all participants or their guardians (for children younger than 18 years old) before enrollment in the study. The investigator was blinded to the group. The study was approved by research ethics committee of Shanghai Ninth People’s Hospital.

### RNA extraction and real-time polymerase chain reaction

The RNAiso Plus reagent (Takara) was used for RNA extraction, and a PrimeScript RT Kit (Takara) was used for reverse transcription or 1.5 μg RNA based on provided protocols. Quantitative real-time reverse transcription polymerase chain reaction (qRT-PCRs) was conducted with the 7500 Fast Real-Time PCR System (Applied Biosystems, Carlsbad, CA). GAPDH was used as a normalization control. Primers sequence were listed in Table S1.

### Western blot

Loading lysis buffer was used to extract total protein from cells. The Lowry protein assay was then used for protein level quantification. 30 mg of protein lysate were then run on an SDS/PAGE gel and transferred to a PVDF membrane (Millipore, Milford, MA), probed with appropriate primary and HRP–conjugated secondary antibodies in a 5% bovine serum albumin solution. Antibodies used were: H3K36me2 (Abways, AY2513), WHSC1 (ActiveMotif, 39880), BCL2 (Abways, AY1364), GAPDH (ABways, AB0036), Cleaved caspase 3 (Cell Signaling Technology, 9664), Ki67(Cell Signaling Technology, 9449).

### CCK-8 asay

The Cell Counting Kit-8 (Beyotime) was used in accordance with provided protocols. Briefly, 2000 cells/well were added to a 96-well plate, 10 ul CCK-8 solution was added, cells were incubated for 2 h, and absorbance was then read.

### Colony formation assay

1000 cells were plated per well in 6-well plates. After 10 days, Crystal Violet Staining Solution (Beyotime, C0121) was used to stain cells and all colonies with 20 or more cells were counted.

### Flow cytometry

1 × 10^5^ stable WHSC1-knockdown and control cells were plated in a 6 well plate. 48 h later, cells were collected for flow cytometry analysis. For cell cycle analysis using a Cell Light Edu Kit (Ruibo, C10313). 7AAD and Annexin-V-PE (BD Biosciences) were used for apoptosis staining.

### Tumorigenesis assay

2 × 10^6^ HCT116 cells that had been transduced with appropriate lentivirus were injected subcutaneously in a total volume of 200 μL into the right flank of nude mice (randomized in two groups). Every 3 days, tumor size was measured. The formula used to assess tumor volume was *V* = 1/2(a*b*b), with *a* being the major and *b* the minor tumor axes. Mice were euthanized after 3–4 weeks, and tumor weight was assessed. The investigator was blinded to the group. Experiments were consistent with the European Community guidelines for the use of experimental animals.

### ChIP-qPCR

1 × 10^6^ CRC cells were crosslinked and lysed. UCD-300 (Bioruptor) was used to shear crosslinked DNA to ~200–1000 base pairs in length. ChIP was performed according to manufacturer’s instructions of Chromatin Immunoprecipitation Kit (17–371; Millipore), followed by qPCR for quantification of ChIP-enriched DNA. The antibodies used for ChIP were anti-H3K36me3 (Active Motif; 61021) and normal mouse IgG (Active Motif; 12-371B). Primers sequence were listed in Table S2.

### Statistical analysis

All data are representative of three independent experiments and all data were shown as mean ± standard deviation (SD). Statistical analyses were performed using R (http://www.r-project.org/), and statistical significance was determined by two-tailed Student’s *t* test or Spearman correlation coefficients test. For all statistical tests, A *P* value < 0.05 was considered that the difference is statistically significant. (*P < 0.05).

## Supplementary information

supplementary Table 1

supplementary Table 2

## References

[1] Keum N, Giovannucci E (2019). Global burden of colorectal cancer: emerging trends, risk factors and prevention strategies. Nat. Rev. Gastroenterol. Hepatol..

[2] Fearon ER (2011). Molecular genetics of colorectal cancer. Annu Rev. Pathol..

[3] Katona BW, Weiss JM (2020). Chemoprevention of colorectal cancer. Gastroenterology.

[4] Baretton GB (1996). Apoptosis and immunohistochemical bcl-2 expression in colorectal adenomas and carcinomas – aspects of carcinogenesis and prognostic significance. Cancer.

[5] Wu DW, Huang CC, Chang SW, Chen TH, Lee H (2015). Bcl-2 stabilization by paxillin confers 5-fluorouracil resistance in colorectal cancer. Cell Death Differ..

[6] Tran TQ, Lowman XH, Kong M (2017). Molecular pathways: metabolic control of histone methylation and gene expression in cancer. Clin. Cancer Res..

[7] Baylin SB, Jones PA (2011). A decade of exploring the cancer epigenome – biological and translational implications. Nat. Rev. Cancer.

[8] Grady WM, Markowitz SD (2002). Genetic and epigenetic alterations in colon cancer. Annu Rev. Genom. Hum. Genet..

[9] Jung G, Hernandez-Illan E, Moreira L, Balaguer F, Goel A (2020). Epigenetics of colorectal cancer: biomarker and therapeutic potential. Nat. Rev. Gastroenterol. Hepatol..

[10] Raskov H, Soby JH, Troelsen J, Bojesen RD, Gogenur I (2020). Driver gene mutations and epigenetics in colorectal cancer. Ann. Surg..

[11] Wang CG (2010). EZH2 and STAT6 expression profiles are correlated with colorectal cancer stage and prognosis. World J. Gastroentero..

[12] Kodach LL (2010). The role of EZH2 and DNA methylation in the silencing of the tumour suppressor RUNX3 in colorectal cancer. Carcinogenesis.

[13] Zhou J (2019). Targeting EZH2 histone methyltransferase activity alleviates experimental intestinal inflammation. Nat. Commun..

[14] Li Y (2009). The target of the NSD family of histone lysine methyltransferases depends on the nature of the substrate. J. Biol. Chem..

[15] Nimura K (2009). A histone H3 lysine 36 trimethyltransferase links Nkx2-5 to Wolf-Hirschhorn syndrome. Nature.

[16] Campos-Sanchez E (2017). Wolf-Hirschhorn syndrome candidate 1 is necessary for correct hematopoietic and B cell development. Cell Rep..

[17] Toyokawa G (2011). Histone lysine methyltransferase Wolf-Hirschhorn syndrome candidate 1 is involved in human carcinogenesis through regulation of the Wnt pathway. Neoplasia.

[18] Yang P (2012). Histone methyltransferase NSD2/MMSET mediates constitutive NF-kappaB signaling for cancer cell proliferation, survival, and tumor growth via a feed-forward loop. Mol. Cell Biol..

[19] Liu C (2019). Knockdown of histone methyltransferase WHSC1 induces apoptosis and inhibits cell proliferation and tumorigenesis in salivary adenoid cystic carcinoma. Anticancer Res..

[20] Ezponda T (2013). The histone methyltransferase MMSET/WHSC1 activates TWIST1 to promote an epithelial-mesenchymal transition and invasive properties of prostate cancer. Oncogene.

[21] Li N (2017). AKT-mediated stabilization of histone methyltransferase WHSC1 promotes prostate cancer metastasis. J. Clin. Investig..

[22] Lindner AU (2013). Systems analysis of BCL2 protein family interactions establishes a model to predict responses to chemotherapy. Cancer Res..

[23] Andre T (2004). Oxaliplatin, fluorouracil, and leucovorin as adjuvant treatment for colon cancer. N. Engl. J. Med..

[24] Roberts AW (2016). Targeting BCL2 with venetoclax in relapsed chronic lymphocytic leukemia. N. Engl. J. Med..

[25] Pollyea DA (2018). Venetoclax with azacitidine disrupts energy metabolism and targets leukemia stem cells in patients with acute myeloid leukemia. Nat. Med..

[26] Jiang M, Milner J (2003). Bcl-2 constitutively suppresses p53-dependent apoptosis in colorectal cancer cells. Br. J. Cancer.

[27] Reed JC (2018). Bcl-2 on the brink of breakthroughs in cancer treatment. Cell Death Differ..

[28] Timucin AC, Basaga H, Kutuk O (2019). Selective targeting of antiapoptotic BCL-2 proteins in cancer. Med. Res. Rev..

[29] Smerage JB (2013). Monitoring apoptosis and Bcl-2 on circulating tumor cells in patients with metastatic breast cancer. Mol. Oncol..

[30] Huang XF, Chen JZ (2009). Obesity, the PI3K/Akt signal pathway and colon cancer. Obes. Rev..

[31] Jhawer M (2008). PIK3CA mutation/PTEN expression status predicts response of colon cancer cells to the epidermal growth factor receptor inhibitor cetuximab. Cancer Res..

[32] Dasgupta M, Dermawan JK, Willard B, Stark GR (2015). STAT3-driven transcription depends upon the dimethylation of K49 by EZH2. Proc. Natl Acad. Sci. USA.

[33] Levy D (2011). Lysine methylation of the NF-kappaB subunit RelA by SETD6 couples activity of the histone methyltransferase GLP at chromatin to tonic repression of NF-kappaB signaling. Nat. Immunol..

