# Correction to: Histone methyltransferase WHSC1 inhibits colorectal cancer cell apoptosis via targeting anti-apoptotic BCL2

**DOI:** 10.1038/s41420-021-00416-0

**Published:** 2021-10-21

**Authors:** Yu Wang, Liming Zhu, Mei Guo, Gang Sun, Kun Zhou, Wenjing Pang, Dachun Cao, Xin Tang, Xiangjun Meng

**Affiliations:** 1 Departments of Gastroenterology, Shanghai Ninth People’s Hospital, Shanghai Jiaotong University School of Medicine, Shanghai, China; 2 Departments of Geriatrics, Shanghai Ninth People’s Hospital, Shanghai Jiaotong University School of Medicine, Shanghai, China

**Keywords:** Colon cancer, Apoptosis, Epigenetics

Correction to: *Cell Death Discovery*

10.1038/s41420-021-00402-6 published online 19 January 2021

The original version of this article unfortunately contained a mistake. The last word of the third sentence in the abstract “OS patients” should be changed to “CRC patients”. The authors apologize for the mistake. The original article has been corrected.

