# Supplementary material for: Histone methyltransferase WHSC1 inhibits colorectal cancer cell apoptosis via targeting anti-apoptotic BCL2
Source: Cell Death Discov. 2021 Jan 19;7:19. doi: 10.1038/s41420-021-00402-6 (PMC7815777; doi:10.1038/s41420-021-00402-6)
Supplement: Supplementary file 1 — supplementary Table 1 [file 41420_2021_402_MOESM1_ESM.docx]

Table S1 Sequences of RT-qPCR primers.

| Gene Name | Forward | Reverse |
| --- | --- | --- |
| WHSC1 | CCACCATACAAGCACAT | TCAGACACTCCGAATCAA |
| GAPDH | TCTGATTTGGTCGTATTGGG | GGAAGATGGTGATGGGATT |
| BCL2 | GTGGCCTTCTTTGAGTTCG | CATCCCAGCCTCCGTTAT |
| BAX | CCCGAGAGGTCTTTTTCCGAG | CCAGCCCATGATGGTTCTGAT |
| BAD | AGGATGAGTGACGAGTTTGTG | CCTGCCCAAGTTCCGATC |
| BCL-xL | GACATCCCAGCTCCACATC | GTTCCCATAGAGTTCCACAAAAG |
| MCL1 | AAGGACAAAACGGGACTGG | ATATGCCAAACCAGCTCCTAC |
| BIM | TGGAGACGAGTTTAACGCTTAC | CCGCAAAGAACCTGTCAATG |
| BID | ATTAACCAGAACCTACGCACC | TGACCACATCGAGCTTTAGC |
| NOXA | GGAGATGCCTGGGAAGAAG | TGCCGGAAGTTCAGTTTGTC |
