# Supplementary material for: Histone methyltransferase WHSC1 inhibits colorectal cancer cell apoptosis via targeting anti-apoptotic BCL2
Source: Cell Death Discov. 2021 Jan 19;7:19. doi: 10.1038/s41420-021-00402-6 (PMC7815777; doi:10.1038/s41420-021-00402-6)
Supplement: Supplementary file 2 — supplementary Table 2 [file 41420_2021_402_MOESM2_ESM.docx]

Table S2 Sequences of ChIP-qPCR primers.

| Primer Name | Forward | Reverse |
| --- | --- | --- |
| CHIP-BCL2-P1 | TATCTTGGAGGCTGGTGT | TTAGCAGAGCGTAGTGGC |
| CHIP-BCL2-P2 | GGTCTCCACCTTTGCCTC | CCAATAATCCAGTGTCCCTA |
| CHIP-BCL2-P3 | CGGACTAGGTGTTCAGGT | CCGTGTATGTGGGAGTGT |
| CHIP-BCL2-P4 | GGTGCCTGTCCTCTTACTT | AGGAGGGCTCTTTCTTTC |
| CHIP-BCL2-P5 | CCAGGAGGAGGAGAAAGG | GGATAAATGAAGGCAGGAC |
| CHIP-BCL2-P6 | TAAGGCAACGATCCCATC | GACTTCTGCGAATACCGG |
| CHIP-BCL2-P7 | TGTATGCCCTGCTTTCAC | CGGTTATCGTACCCTGTTC |
| CHIP-BCL2-P8 | CCACAGGGCGATGTTGTC | GAGTGGGATGCGGGAGAT |
